# To what extent do older adult community exercise programs in Winnipeg, Canada address balance and include effective fall prevention exercise? A descriptive self-report study

**DOI:** 10.1186/s12877-019-1224-x

**Published:** 2019-07-29

**Authors:** Kathryn M. Sibley, Alexie J. Touchette, Jonathan C. Singer, Kathleen M. A. Dubberley, Alison R. Oates

**Affiliations:** 1 Department of Community Health Sciences Rady Faculty of Health Sciences, University of Manitoba, 379- 753 McDermot Avenue, Winnipeg, MB R3E 0W3 Canada; 2 George and Fay Yee Centre for Healthcare Innovation, 379- 753 McDermot Avenue, Winnipeg, MB R3E 0W3 Canada; 3 Faculty of Kinesiology and Recreation Management, University of Manitoba, Winnipeg, MB Canada; 4 Winnipeg Regional Health Authority, 650 Main Street, Winnipeg, MB Canada; 5 College of Kinesiology, University of Saskatchewan, 87 Campus Drive, Saskatoon, SK Canada

**Keywords:** Postural balance, Accidental falls, Aging, Evidence-based, Guidelines, Recommendations

## Abstract

**Background:**

Effective fall prevention exercise for community-dwelling older adults requires (i) challenging balance exercise, (ii) offered at least 3 hrs/ week, and (iii) on an ongoing basis, to reduce falls. Community exercise programs are a potential implementation strategy for fall prevention exercise; however, the extent to which they address balance and include effective fall prevention exercise is unknown. Study objectives were to describe program delivery, exercise design, and assessment characteristics of older adult community exercise programs in Winnipeg, Canada; determine if they included effective fall prevention exercise; determine the balance challenge and components of postural control addressed in the most- and least-frequently reported exercises.

**Methods:**

A public inventory of older adult community exercise programs served as the sampling frame for cross-sectional telephone questionnaires exploring program, exercise, and assessment characteristics. Exercises were coded independently by two investigators for balance challenge level and components of postural control. Programs were categorized by number of effective fall prevention exercise components established by evidence-based recommendations. Descriptive statistics were calculated.

**Results:**

Thirty-three eligible programs were identified and nine individuals participated. Most programs (*n* = 5, 56%) identified as general exercise, and two (22%) as fall prevention exercise. Most programs (n = 5, 56%) were offered two or more times/ week and reported exercise intensity as somewhat challenging. Exercise time offered ranged between 1 and 3 h/ week. Assessments were conducted in two programs (22%). Only one program (general exercise) included all components of effective fall prevention exercise. Two programs (22%) included the component of being offered at least 3 hrs/ week. Three programs (33%) included the component of being offered on an ongoing basis. Seven programs (78%) prescribed mostly moderate challenge balance exercise, and one program (11%) prescribed mostly high challenge exercise. Most of the 19 most-frequently prescribed exercises (*n* = 17, 89%) targeted static stability and none targeted reactive postural control.

**Conclusions:**

Most of the older adult community exercise programs participating in this study did not focus on fall prevention, and did not include all components of effective fall prevention exercise. Future studies should focus on fall prevention programs and explore factors influencing implementation of effective fall prevention exercise components to facilitate planning.

**Electronic supplementary material:**

The online version of this article (10.1186/s12877-019-1224-x) contains supplementary material, which is available to authorized users.

## Background

The global population is aging at an unprecedented rate [1]. Among many threats to successful aging, falls are a major concern with advancing age. The common definition of a fall [2] – an event which results in a person coming to rest inadvertently on the ground, floor or other lower level – does not reflect its potential severity. Too often, a fall is the catalyst for a downward health spiral that is associated with injury [3, 4], hospitalization [5], long term care admission [6], and even death [7].

There is a substantial body of peer-reviewed research studying interventions to reduce falls and their associated consequences, and synthesis of this evidence has identified that exercise is a critical component of effective fall prevention interventions. A 2017 systematic review of 283 fall prevention interventions in older adults across all settings included a network meta-analysis comparing 25 different intervention components [8]. Exercise was a feature in three of four intervention combinations found to significantly reduce injurious falls. The authors concluded that exercise was likely the most effective intervention for reducing the number of fallers, injurious falls, fractures, and hip fractures. This comprehensive finding supports multiple meta analyses demonstrating that exercise is an effective fall prevention intervention for community-dwelling older adults [9, 10].

Key components of exercise design that should be included in effective fall prevention exercise have been identified. In 2017, Sherrington et al. reviewed 99 fall prevention exercise interventions for community-dwelling older adults and conducted sub-groups analyses to identify characteristics of effective exercise programs [11]. They determined that overall, exercise reduced the rate of falls by 21%, with greater reductions among programs that included challenging balance exercise and more than 3 hrs of exercise per week. Based on this evidence, practice recommendations were proposed. To reduce falls, they include recommending (i) challenging balance exercise which involves reducing the base of support, moving the centre of mass, and controlling body position during standing, and reducing reliance on arms for support, (ii) for at least 3 hrs per week, (iii) on an ongoing basis. It is also well-recognized that balance is complex, presenting as distinct components resulting from interactions between multiple biologic systems and the environment [12–14]. These distinct components include: static stability during standing, dynamic stability during walking, anticipatory adjustments prior to discrete voluntary movements, postural reactions to recover stability, orienting relative to gravity, moving the center of mass to the edge of the base of support, integrating sensory information, and influence of cognitive processing on the maintenance of stability [14, 15]. Comprehensive assessment and treatment approaches that address multiple components of balance using this systems perspective are also recommended [15].

Ensuring effective fall prevention exercise is available for older adults is one critical factor necessary for achieving real-world effectiveness in widespread practice [16]. Community exercise programs – ongoing physical activity classes offered in non-healthcare settings that aim to promote health and are accessible through public or private funding [17] – represent a cornerstone of healthy living strategies [18, 19] for addressing global physical activity recommendations [20]. Such programs have the potential to influence the health of many older adults because of their collective reach and accessibility to a significant proportion of the population. As such, if the structure of community exercise programs included effective fall prevention exercise, there could be a meaningful influence on fall outcomes. However, data exploring the extent to which community exercise programs for older adults address balance and include the exercise design components proven to effectively reduce falls is sparse. Understanding older adult community exercise programs exercise components, and specifically, if and how balance and effective fall prevention exercise are incorporated, is critical for exploring their potential as a real-world fall prevention implementation strategy. As a key first step, implementation process models emphasize the importance of understanding current practice [16], which are then used to inform the development of research, education and policy initiatives to optimize best practice.

The overall goal of this study was to establish a baseline understanding of the extent to which older adult community exercise programs in Winnipeg, Canada addressed balance and included effective fall prevention exercise. Winnipeg is the capital and largest city [population 749,500 [21]] in Manitoba, Canada’s 5th largest province [22]. Falls are the leading cause of injury-related hospitalizations (86%) among older adults residing in the Winnipeg health region, where there are on average 1,647 older adults hospitalized per year for a fall [23]. Falls are also the leading cause of injury deaths for older adults, accounting for 90% of fall deaths for Winnipeg residents of all ages. In 2015, Manitoba reported the highest provincial rate of fall-related mortality in Canada [5]. The objectives of this study were to (i) describe program delivery, exercise design, and assessment characteristics of older adult community exercise programs in Winnipeg; (ii) determine the extent to which programs included effective fall prevention exercise according to evidence-based recommendations; and (iii) determine the balance challenge and components of postural control addressed in the most- and least-frequently reported exercises.

## Methods

### Study design

A cross-sectional self-report study was conducted in 2016 through a survey questionnaire administered by telephone interview following a modified Dillman recruitment approach [24]. The Checklist for Reporting Results of Internet E-Surveys recommendations for survey conduct and reporting [25] were adopted as appropriate. Ethics approval was obtained from the University of Manitoba health research ethics board.

### Sampling frame

Community exercise programs targeting older adults (broadly characterized as those aged 50 years and older) living independently in non-institutional settings in Winnipeg, Manitoba were eligible for this study. The sampling frame was based on an existing inventory of Winnipeg community organizations offering exercise programs for older adults established by the Winnipeg Regional Health Authority public health injury prevention program in 2016. The inventory was developed by the health authority through an environmental scan with the intention of identifying and recommending fall prevention programs. Inventory inclusion criteria established by the health authority were based on 2011 fall prevention exercise guidelines [26], and included programs identified as spending at least 40% of the program on weight-bearing exercise that challenged strength and/ or balance and were designed and supervised by a trained instructor or healthcare professional. As the program characteristics were not independently verified, for the purpose of this study, the inventory programs were considered as having a minimum baseline potential for fall prevention only. If an organization offered multiple exercise programs for older adults, the program most directly related to fall prevention (either through a stated objective or established systematic review evidence as an effective intervention [Additional file 1] was considered for the present study. Thirty-three programs were identified as eligible for this study.

### Participants

Participants were individuals identified as having a strong knowledge of the older adult exercise program under study; in most cases a director, manager, or instructor. The specific individual who was considered most appropriate to complete the survey questionnaire was decided by the organization during the recruitment process. Written informed consent was obtained from all individual participants, with institutional support from their organization.

### Recruitment

An invitation message was emailed to eligible organizations through publicly-available contact information. The message was sent to the program director when possible or to the general information account from the Principal Investigator’s institutional email address. The invitation message introduced the study, asked the recipient to forward the message to the most appropriate individual to complete the survey if necessary, and reply if interested to receive more information and/ or participate in the study. Reminder messages were sent weekly for two weeks if no response was received. On receipt of a reply expressing interest in the study, eligibility was confirmed and consent documents were provided. If consent was received, the older adult exercise program most directly related to fall prevention was determined, and an interview time was scheduled. The questionnaire was distributed to participants prior to the interview.

### Questionnaire instrument

Given the absence of existing instruments for determining community exercise practices, a custom questionnaire was developed for the study [Additional file 2]. The questionnaire document and interview format underwent pilot testing for sensibility, and face and content validity with a convenience sample of primary instructors from three older adult exercise programs. Pilot data were not included in the final analysis. Questionnaire items included closed- and open-ended questions. Variables in the questionnaire included characteristics of the program and its delivery (e.g. goals and objectives, perceived focus on fall prevention and balance), participant population characteristics (e.g. target population age range, health status, inclusion criteria), exercise design characteristics [e.g. frequency, intensity, time, and type of balance exercise [27]], assessment practices and instruments used, and characteristics of the individual completing the interview (e.g. role, educational background, years of experience).

### Data collection, processing and analysis

The questionnaire was administered through a structured telephone interview scheduled at the participants’ convenience. Telephone administration was chosen to facilitate response of the open-ended questions and ease participant burden. During the interviews, a research assistant read each question and response options (if a closed-ended question) and manually completed the survey as dictated by participants using into online survey software (Fluid Surveys). Interviews were audio-recorded. Offline, survey responses were downloaded to a Microsoft Excel spreadsheet.

Open-ended questions were reviewed and coded into categories using Microsoft Excel by a research assistant and then reviewed by the Principal Investigator. Exercises were coded by level of balance challenge and component of balance targeted. Two investigators independently coded each exercise and disagreements were resolved by review, discussion, and consensus with a third investigator. Level of balance challenge was operationalized into a five-point summary score based on the 2017 evidence-based practice recommendations [11]. Points were assigned based on arm challenge (0 = supported, 1 = unsupported), base of support challenge (0 = minimal, 1 = some, 2 = significant), and centre of mass challenge (0 = minimal, 1 = some, 2 = significant). Balance components were coded using nine published operational definitions [28] [Additional file 3]. For the purpose of this study considering typical community-dwelling older adults, a total balance challenge score less than or equal to 1 was considered “low”, scores equalling 2 or 3 were considered “moderate”, and scores equalling 4 or greater were considered “high”. The extent to which program characteristics included effective fall prevention exercise components was determined by calculating (i) if the program was offered at least 3 h per week; (ii) if the program was offered on an ongoing basis; and, (iii) whether the majority (> 50%) of balance exercises prescribed in a program were classified as low, moderate, or high challenge. The balance challenge scores and components of postural control for the most-commonly prescribed exercises (defined as those reported by more than two-thirds of participants) were compared to the components and challenge level of the least-commonly prescribed exercises (defined as those reported by less than one-third of participants). Quantitative analysis was performed with SPSS v25. Data were summarized using descriptive statistics (frequencies, proportions, range and median as appropriate).

## Results

### Recruitment and participant characteristics

**Fig. 1:**
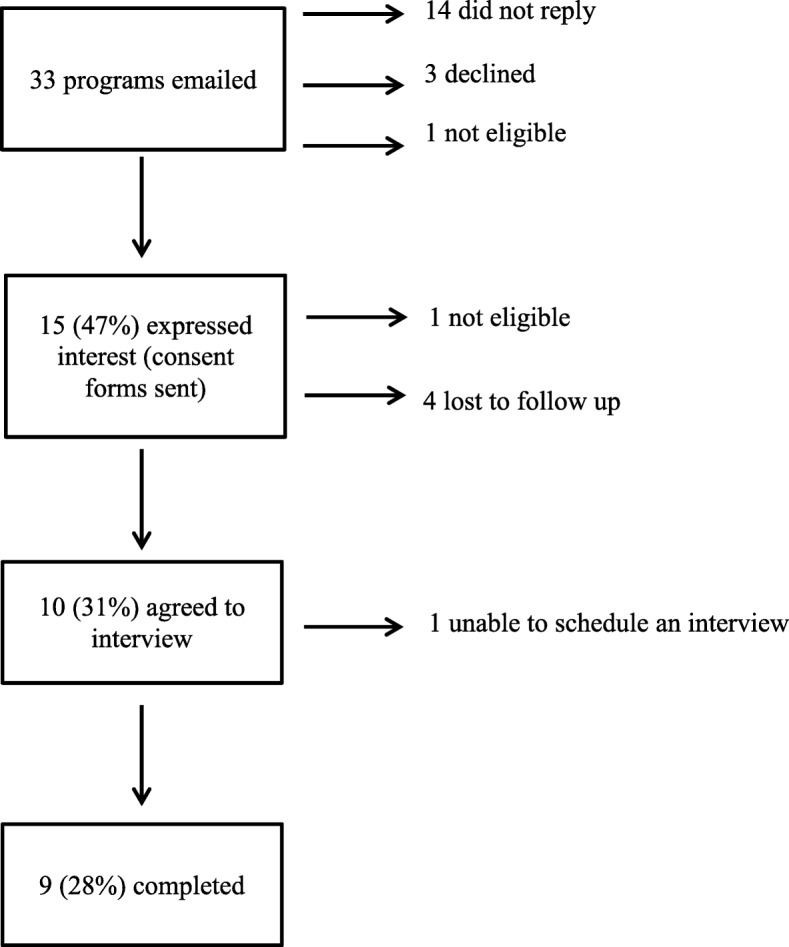
Study flow diagram

Recruitment flow is illustrated in Fig. 1. Thirty-three organizations with an eligible older adult exercise program were identified and invited. Individuals from 15 organizations (47%) expressed interest in the study. Ten individuals (31%) agreed to participate; however, one was lost to follow-up. Reasons for non-participation were not obtained. Questionnaire interviews were completed for nine programs (28%). Most participants were either an exercise leader or program director (*n* = 6, 67%), and had more than 5 years experience in their role (*n* = 7, 78%). Participants reported a range of educational backgrounds, and the majority reported receiving some additional training in fall prevention (*n* = 6, 67%).

### Program delivery, exercise design, and assessment characteristics (Table 1)

Overall program goals and objectives described by participants centered on enhancing movement and activity (*n* = 6, 67%), improving physiological performance (*n* = 7, 78%), and promoting psychological well-being (*n* = 5, 56%). Five participants (56%) reported that their program was either somewhat or very focused on balance. The majority of participants (*n* = 6, 67%) reported their programs were somewhat focused on fall prevention. Estimated client age range spanned from older adults in their 50’s (*n* = 1, 11%) to 80’s (*n* = 5, 56%). All nine participants estimated their program included older adult clients with an approximate age in their 70’s. Most participants reported that their programs did not target a specific older adult population (*n* = 8, 89%), nor had any specific inclusion criteria (*n* = 5, 56%).

**Table 1:** Program delivery, client and exercise design characteristics

Characteristic		Number of programs	Percent of programs
Estimated client age range	50’s	1	11%
	60’s	3	33%
	70’s	9	100%
	80’s	5	56%
Specific older adult population targeted	No	8	89%
	Yes	1	11%
	Healthy older adults		
Specific inclusion criteria	No	5	56%
	Yes	4	44%
	Minimum independence level	3	33%
	Minimum strength level	3	33%
	Medical clearance	3	33%
Balance focus level	A little	4	44%
	Somewhat	2	22%
	Very	3	33%
Fall prevention focus level	Somewhat	6	67%
	Significant	3	33%
Exercise frequency (# classes/week)	1 or less	4	44%
	2 or more	5	56%
Exercise intensity (perceived challenge)	Easy	1	11%
	Somewhat	5	56%
	Challenging	3	33%
Exercise time (total hours/week)	1	4	44%
	1.2	3	33%
	3	2	22%
Exercise type	General exercise	5	56%
	Fall prevention	2	22%
	Tai Chi (any form)	1	11%
	Strength	1	11%

With regards to the frequency of exercise, the majority of participants (n = 5, 56%) reported their programs were offered two or more times per week. Three participants (33%) reported that their programs provided a handout describing home-based exercises, and one of these three also distributed an exercise log. With regards to intensity of exercise, the majority of participants (n = 5, 56%) estimated that their programs were somewhat challenging for clients. When probed about how exercise intensity/challenge was determined, most participants (*n* = 7, 78%) reported that challenge was modified based on successful performance of prescribed tasks. Other factors influencing balance challenge prescription included overall health of client (*n* = 2, 22%), time-based progression in class (n = 2, 22%), doctor or physiotherapist prescription, initial assessment, client choice, and understanding of exercise design (all *n* = 1, 11%). Time of exercise offered ranged between one and 3 hrs per week, with most participants (*n* = 4, 44%) reporting a total of 1 hr of exercise was offered per week. The majority of participants (*n* = 6, 67%) reported that their programs were offered for a fixed duration, mostly either 3 or 4 sessions per year ranging from 6 to 12 weeks. Three participants (33%) reported that their programs were offered on an ongoing basis. With regards to type of exercise, five participants (56%) described their program as a general exercise class, two participants (22%) described their program as a fall prevention class, one (11%) identified as tai chi, and one (11%) as a strength exercise class. Nineteen exercises were prescribed by more than two-thirds of programs, and 11 exercises were prescribed by less one-third of programs.

Two participants (22%) reported that their programs conducted standardized assessments at the beginning and end of the program. Both of these were the programs identified as fall prevention exercise. The measures used included the Berg Balance Scale [29] (*n* = 2, 22%), Fullerton Advanced Balance Scale [30], Timed Up-and-Go Test [31], 30s Sit-to-Stand (all *n* = 1, 11%).

### Effective fall prevention exercise components

Based on the information provided by participants, two programs (22%) included the component of being offered for at least 3 hrs per week. Three programs (33%) included the component of being offered on an ongoing basis. Regarding the balance challenge component, one program (11%) prescribed mostly low challenge balance exercise, seven programs (78%) prescribed mostly moderate challenge balance exercise, and one program prescribed mostly high challenge exercise. The majority of programs (*n* = 8, 89%) included one or two effective fall prevention exercise components, while one program (11%) included all three components. The program that included all three effective fall prevention exercise components identified as general exercise. The two programs that identified as fall prevention both prescribed mostly moderate challenge balance exercise, but were not offered at least 3 hrs per week or offered on an ongoing basis.

### Balance challenge and components of postural control targeted

Among the 19 exercises reported by more than two-thirds of participants (Table 2), the median challenge score was 1 (out of 5). The majority of these exercises (*n* = 17, 89%) targeted static stability. Less than one third of the exercises targeted any of the other balance components, and none of these exercises targeted reactive postural control or cognitive contributions. Among the 11 exercises reported by less than one-third of participants (Table 3), the median challenge score was 4 (out of 5). Most of these exercises targeted dynamic stability (*n* = 10, 91%) and anticipatory postural control (*n* = 8, 73%), as well as cognitive contributions (*n* = 6, 55%). None of these exercises targeted static balance, and just one targeted reactive postural control.

**Table 2:** Most-frequently reported exercises (*n* = 19)

Exercise	Number of programs	Challenge score (max = 5)	Component of postural control targeted							
			Functional stability limits	Static stability	Dynamic stability	Reactive postural control	Anticipatory postural control	Dynamic stability	Sensory contributions	Cognitive contributions
Supported standing with eyes closed	9	0	No	Yes	Yes	No	No	No	Yes	No
Supported standing narrow stance	9	1	No	Yes	No	No	No	No	No	No
Supported shifting weight as far as possible in either direction	9	1	Yes	Yes	Yes	No	Yes	No	No	No
Unsupported basic standing focused on not leaning/ staying upright relative to floor/gravity	9	1	No	Yes	Yes	No	No	No	No	No
Standing unsupported with eyes closed	9	1	No	Yes	Yes	No	No	No	Yes	No
Raising arm - any direction	9	2	No	Yes	No	No	Yes	No	No	No
Shifting weight as far as possible in either direction	9	3	Yes	Yes	Yes	No	Yes	No	No	No
Ankle strategy, weight shifts	9	3	Yes	Yes	No	No	No	No	No	No
Heel raises	9	4	No	No	No	No	Yes	Yes	No	No
Supported standing, tandem stance	8	1	No	Yes	No	No	No	No	No	No
Supported one-legged stance	8	1	No	Yes	No	No	No	No	No	No
Hip strategy, weight shifts	8	3	Yes	Yes	No	no	No	No	No	No
Sit to stand without hands	8	4	No	No	No	No	Yes	Yes	No	No
Supported basic standing, focused on not leaning/ staying upright relative to floor/ gravity	7	0	No	Yes	Yes	No	No	No	No	No
Supported basic standing comfortable position	7	0	No	Yes	No	No	No	No	No	No
Standing supported wide stance	7	0	No	Yes	No	No	No	No	No	No
Unsupported standing narrow stance	7	2	No	Yes	No	No	No	No	No	No
Unsupported standing tandem stance	7	3	No	Yes	No	No	No	No	No	No
Unsupported one-legged stance	7	3	No	Yes	No	No	No	No	No	No

**Table 3:** Least-frequently reported exercises (*n* = 17)

Exercise	Number of programs	Challenge score (max = 5)	Component of postural control targeted							
			Functional stability limits	Static stability	Dynamic stability	Reactive postural control	Anticipatory postural control	Dynamic stability	Sensory contributions	Cognitive contributions
Supported Standing on unstable surface (while supported)	3	1	No	Yes	Yes	Depends^a^	No	Yes	No	No
Walking with head turns	3	3	No	No	No	No	Yes	Yes	Yes	Yes
Walking while balancing object	3	3	No	No	No	No	Yes	Yes	No	Yes
Obstacle courses	3	4	No	No	No	No	Yes	Yes	No	Yes
Walking and picking up objects	3	4	No	No	No	No	Yes	Yes	No	Yes
Standing on unstable surface such as wobble board,mat, etc.	3	5	No	Yes	Yes	Depends^a^	No	Yes	No	No
Supported Toe taps on bench step - any direction	2	2	No	No	No	No	Yes	Yes	No	Yes
Walking (fast pace) extended - cardio (2 min)	2	3	No	No	No	No	Yes	Yes	No	No
Toe taps on bench step - any direction	2	4	No	Yes	No	No	Yes	Yes	No	Yes
Pushing /nudging/ perturbing/ throwing off balance	2	4	No	Depends^a^	Yes	Yes	No	Depends^a^	No	No
Stair walking	0	5	No	No	No	No	Yes	Yes	No	No

^a^Balance component targeted depends on the unstable surface used in the exercises, and response of the participant

## Discussion

To our knowledge, this is the first comprehensive examination of balance and fall prevention exercise content in a sample of older adult community exercise programs. In spite of the small sample size and self-reported data, the findings offer insight regarding some older adult community exercise programs in Winnipeg, Canada and inclusion of evidence-based effective fall prevention exercise.

An important finding was the observation that most of the older adult community exercise programs participating in this study did not explicitly focus on fall prevention. The goals of the programs as described by participants focused on more general health and social benefits. To this end, a second important finding was the limited inclusion of effective fall preveniton exercise in most of the community exercise programs described in the present study. This mismatch is perhaps not surprising given the low focus on fall prevention in this sample. Regardless, these findings have implications when considering the potential effectiveness of existing community exercise programs for fall prevention, in light of the specific nature of exercise required to reduce fall risk. For example, analysis of the postural control systems targeted in the most-commonly reported exercises revealed that some components were infrequently addressed. With respect to fall prevention this is concerning as it is well-established that some of these under-targeted components, such as reactive balance, have an important and specific role in avoiding falls [32]. While there is little published data on community exercise program practices to compare against, these findings are consistent with related work in physical therapists that demonstrated that reactive balance is consistently under-assessed in Canada [33, 34]. Finally, a third important finding was the general absense of assessment in the programs discussed in this study. This is important because assessment is recognized for addressing the need to tailor exercise prescription to specific abilities and monitor change over time [15].

Arguably the most significant findings related to the absense of effective fall prevention exercise content in the two self-identified fall prevention exercise programs, neither of which included challenging balance exercise offered for 3 hrs per week on an ongoing basis. It is beyond the scope of the study findings to speculate on the underlying rationale shaping the exercise design characteristics of the older adult community exercise programs included in the present study. However, related analyses exploring challenges in implementing evidence-based balance and fall prevention exercise in physical therapy practice have illuminated challenges associated with meeting reccomendations in resource-scarce funding climates [35]. A critical next step will be to identify and understand factors influencing program design and delivery in community settings. The potential barriers and facilitators influencing implementation of fall prevention interventions are numerous and complex [36]. Moving forward, implementation determinant frameworks will be essential to guide future analyses of barriers and facilitators influencing implementation of fall prevention exercise in older adult community exercise programs. One such framework is the Consolidated Framework for Implementation Research [37]. This highly-used framework [38] considers five key domains including i) characteristics of the intervention under consideration; ii) the economic, social and political environment in which an organization using the intervention exists (the “outer setting”); iii) the structural, cultural and political conditions of the organization itself (the “inner setting”); iv) characteristics of the individuals adopting an intervention; and, v) features of the implementation process. A comprehensive approach will be essential to identify key issues to inform implementation intervention planning and delivery to ensure effective and sustainable fall prevention exercise is offered in community settings. It is also important to recognize that efforts to implement evidence-based fall prevention exercise service delivery need to be complemented by understanding and incorporating exercise adoption and adherence behaviors among older adults themselves. While beyond the scope of the current project, future studies could incorporate these distinct bodies of work (e.g. [39]) to optimize real world uptake of fall prevention exercise to achieve the ultimate goal of reducing falls.

### Limitations

The primary limitations of the study methods were the limited scope of the sampling frame (restricted to one mid-size Canadian city and existing health authority inventory), low recruitment rate and small final sample size (just nine programs represented), self-reported nature of survey data which may have been open to variations in interpretation and over-estimate the frequency of clinical behavior [40], as well as the coding framework for balance challenge and components of postural control in prescribed exercises. Continued work to expand understanding of current fall prevention exercise practices across jurisdictions (regionally, provincially, nationally and internationally), as well as settings (urban and rural) will be critical to gain an accurate picture of community fall prevention exercise efforts for older adults. To address limitations of self-report, in-depth longitudinal observation of program content and delivery could also offer reliable and important insight. With regards to the analysis, limitations regarding the coding framework for balance exercises using a systems perspective have been previously identified [28, 41]. While the addition of the balance challenge coding framework in this study addressed some of these issues and strengthens previous analyses, some coding gaps persisted. For example, the balance challenge framework was based on existing recommendations but did not consider how sensory manipulations modify balance challenge. Reduction of sensory input (such as removing vision) is well-established as affecting center of mass control and generally accepted as an indicator of a higher level of balance challenge [42], but this was not reflected in the challenge scores for these types of exercises in the current study. We still lack a comprehensive framework for understanding, considering and measuring the complex nature of balance. Lastly, it is acknowledged that this project was limited to the consideration of only one potential fall prevention exercise delivery method (community exercise programs), and other emerging models for delivering exercise (such as home-based technologies [43, 44] and/ or lifestyle integration approaches [45]) have also demonstrated potential.

## Conclusions

This study provides important data on older adult community exercise programs in real-world settings. The findings have implications for considering the potential of community exercise programs as effective fall prevention interventions. Most older adult community exercise programs described in the current study did not meet include effective fall prevention exercise, and suggest that existing programs are not sufficiently specific if fall prevention is a goal. As the small sampling frame may not be representative of all jurisdictions, the methodology developed here can be applied and expanded to larger regions. The coding frameworks used in the present study may also be useful for critically analyzing exercise content in future work with additional conceptual refinements. Moving forward, we recommend that future consideration of fall prevention exercise in community settings should focus specifically on programs with an explicit fall prevention goal. Next steps should also explore factors influencing the implementation of evidence-based fall prevention exercise to facilitate decision-making and planning at the program and policy levels.

## Additional file

Additional file 1 Priority of type of exercise programs to be included. Doc (DOCX 19 kb)

Additional file 2 Telephone Questionnaire.Doc (DOCX 51 kb)

Additional file 3 Components of balance operational definitions. Doc (DOCX 17 kb)

## Data Availability

The datasets used and analysed during the current study are available from the corresponding author on reasonable request.
