# Supplementary material for: To what extent do older adult community exercise programs in Winnipeg, Canada address balance and include effective fall prevention exercise? A descriptive self-report study
Source: BMC Geriatr. 2019 Jul 29;19:201. doi: 10.1186/s12877-019-1224-x (PMC6664743; doi:10.1186/s12877-019-1224-x)
Supplement: Supplementary file 1 — Priority of type of exercise programs to be included. Doc (DOCX 19 kb) [file 12877_2019_1224_MOESM1_ESM.docx]

**Additional file 1: Priority of type of exercise programs to be included**

| **Priority** | **Type** | **Rationale** | **Reference** |
| --- | --- | --- | --- |
| 1 | “Fall prevention” designation | If specifically identifying as a fall-prevention class, critical to understand what is being offered |  |
| 2 | Tai chi | Systematic Review (SR) evidence of decreased risk of falls and improved balance | Gillespie et al., 2012; Howe et al., 2011 |
| 3 (counter-balanced) | Yoga | SR evidence of improved balance | Howe et al., 2011; Youkhana et al., 2016 |
| 3 (counter-balanced) | Dance | SR evidence of improved balance | Howe et al., 2011; Hwang & Braun, 2015 |
| 3 (counter-balanced) | Strength | SR evidence of improved balance | Howe et al., 2011 |
| 4 | General exercise | SR evidence that General physical activity has not effect on falls, but difficult to categorize exactly what is in this- could have multiple elements that have shown to be effective, or could not. As a screener, this would be lowest priority | Gillespie et al., 2012 |
| 5 | Other, specified by participant |  |  |

**References**

Gillespie LD, Robertson MC, Gillespie WJ, Sherrington C, Gates S, Clemson LM, et al. Interventions for preventing falls in older people living in the community. The Cochrane database of systematic reviews. 2012(9):Cd007146.

Howe TE, Rochester L, Neil F, Skelton DA, Ballinger C. Exercise for improving balance in older people. The Cochrane database of systematic reviews. 2011(11):Cd004963.

Youkhana S, Dean CM, Wolff M, Sherrington C, Tiedemann A. Yoga-based exercise improves balance and mobility in people aged 60 and over: a systematic review and meta-analysis. Age Ageing. 2016;45(1):21-9.

Hwang PW, Braun KL. The Effectiveness of Dance Interventions to Improve Older Adults' Health: A Systematic Literature Review. Alternative therapies in health and medicine. 2015;21(5):64-70.
