# Supplementary material for: To what extent do older adult community exercise programs in Winnipeg, Canada address balance and include effective fall prevention exercise? A descriptive self-report study
Source: BMC Geriatr. 2019 Jul 29;19:201. doi: 10.1186/s12877-019-1224-x (PMC6664743; doi:10.1186/s12877-019-1224-x)
Supplement: Supplementary file 2 — Telephone Questionnaire.Doc (DOCX 51 kb) [file 12877_2019_1224_MOESM2_ESM.docx]

Additional file 2: Telephone Questionnaire

*Thank you for agreeing to participate in this study. The goal of this research is to better understand the nature of ongoing older adult exercise programs in Winnipeg relating to fall prevention. Your responses are highly valued and will contribute to advancing best research and practice in Manitoba and across Canada.*

These questions are for gathering information about the _____________ exercise program offered at your facility.

1. Please describe your program’s goals.

|  |
| --- |
|  |

1. To what extent is fall prevention a focus of your older adult exercise program?
   - 1. Not at all
     2. A little
     3. Somewhat
     4. Significantly
     5. Don’t know
2. Do you track or monitor falls in your participants?
   - 1. No
     2. Yes, specify method(s):__________________________________________
3. To what extent is balance a focus of your older adult exercise program?
   - 1. Not at all
     2. A little (1-25% of program)
     3. Somewhat (25-80% of program)
     4. Very (60-85% of program)
     5. Exclusively (85+% of program)
     6. Don’t know

**Program development**

1. How was the program developed?

|  |
| --- |

1. Who designed the program?

|  |
| --- |
|  |

**Program execution**

1. Who delivers the program?
   - 1. An exercise physiologist/ kinesiologist
     2. A physiotherapist
     3. An exercise leader/ fitness professional
     4. A volunteer/ peer leader
     5. Other, please specify __________________________________
2. Which of the following tasks are involved in delivering the program?
   - 1. administering outcome measure for the client / program
     2. developing the exercise routines
     3. individual consulting
     4. providing home programs
     5. other e.g. equipment set up, etc
     6. Other: please describe _________________________________________
3. What is the participant to instructor ratio? __________________________________
4. Is there an unsupervised portion (e.g.: home exercises)?
5. No
6. Yes
7. If yes: Do you prescribe/send anything home?
   - - - 1. No
         2. Yes
8. If yes, explain:
9. paper
10. handout
11. exercise equipment (e.g., bands, tubing, etc.)
12. exercise log or diary
13. other: _____________________________________________

**Target population, inclusion/exclusion criteria**

1. What is the approximate age of most participants in your program?
2. 50's
3. 60's
4. 70's
5. 80+
6. Does your program focus on any specific older adult population?
   - - 1. No
       2. Specific health conditions,
7. *specify*: ____________________________
   - - 1. cultures/ethnicity
       2. religion
       3. Gender
       4. Other, specify: ____________________________________________
8. What language is the program delivered in? _______________________
9. Are there specific inclusion and exclusion criteria?
   - - 1. No
       2. Yes, please specify: _____________________________________

**Program Design**

1. How many classes are conducted a week?
   - 1. One or less
     2. 2 per week
     3. 3 per week
     4. 4 per week
     5. 5 or more per week
2. How challenging are the exercises for the clients?
   - 1. Not at all
     2. Easy
     3. Somewhat challenging
     4. Challenging
     5. Very challenging (clients need direct supervision)
3. How do you determine how challenging the exercises are for the clients? (e.g.: individual/general)?

|  |
| --- |
|  |

1. Are exercises ever progressed or regressed during later classes?
   - 1. Yes
     2. No
2. Is the challenge prescribed the same for everyone?
   - 1. Yes
     2. No
3. If No, is it tailored to each individual’s ability?
   - - 1. Yes
       2. No, explain: ______________________________________
4. How long is each class, in minutes? ________________________________________
5. In which month and year did your program start? _____________________________
6. Is the program duration ongoing or of a fixed duration?
   - 1. Ongoing
     2. Fixed: How long is it offered for? _______________________________
7. Do you track attendance?
   1. No
   2. Yes, if so what are your program’s attendance rates _______________
8. Could you please provide a general description of the exercises performed in your program?

|  |
| --- |

*“Now I’m going to go through a specific list of exercises. For each, please respond*

| **CATEGORY/ EXERCISE** | **No – not prescribed** | **Yes- sometimes** | **Yes- most of the time** | **IF YES,** | |
| --- | --- | --- | --- | --- | --- |
|  |  |  |  | **Some participants perform** | **Most participants perform** |
| 1. **Standing Balance with Support (holding on)** |  |  |  |  |  |
| Basic standing, focused on not leaning/staying upright relative to floor/gravity |  |  |  |  |  |
| Basic standing comfortable position |  |  |  |  |  |
| Standing wide stance |  |  |  |  |  |
| Standing narrow stance |  |  |  |  |  |
| Standing tandem (toe-heel in front of one another) |  |  |  |  |  |
| One-legged stance |  |  |  |  |  |
| Standing on unstable surface SPECIFY SURFACE |  |  |  |  |  |
| Shifting weight as far as possible in either direction, |  |  |  |  |  |
| Standing with eyes closed |  |  |  |  |  |
| Toe taps on bench step – any direction |  |  |  |  |  |
| 1. **Standing Balance Unsupported** |  |  |  |  |  |
| Basic standing comfortable position |  |  |  |  |  |
| Standing wide stance |  |  |  |  |  |
| Standing narrow stance |  |  |  |  |  |
| Standing tandem toe-heel in front of one another |  |  |  |  |  |
| One-legged stance |  |  |  |  |  |
| Standing on unstable surface (wobble board/mat/etc.)  SPECIFY SURFACE: |  |  |  |  |  |
| Basic standing focused on not leaning/staying upright relative to floor/gravity |  |  |  |  |  |
| Shifting weight as far as possible in either direction, |  |  |  |  |  |
| Standing with eyes closed |  |  |  |  |  |
| Toe taps on bench step – any direction |  |  |  |  |  |
| 1. **Balance/ stability exercises** |  |  |  |  |  |
| Raising arms – any direction |  |  |  |  |  |
| Heel raises |  |  |  |  |  |
| Hip strategy, weight shifts |  |  |  |  |  |
| Ankle strategy, weight shifts |  |  |  |  |  |
| Sit to stand (up from chair) – with hands |  |  |  |  |  |
| Sit to stand (up from chair) – without hands |  |  |  |  |  |
| Obstacle courses (DESCRIBE IN NOTES) |  |  |  |  |  |
| Pushing/nudging/perturbing/throwing off balance |  |  |  |  |  |
| Throwing/catching ball or other projectile |  |  |  |  |  |
| 1. **Walking exercises** |  |  |  |  |  |
| Walking (comfortable pace) |  |  |  |  |  |
| Walking (fast pace) for short duration (10 meters) |  |  |  |  |  |
| Walking (fast pace) extended – cardio (2 minutes) |  |  |  |  |  |
| Walking on toes |  |  |  |  |  |
| Walking on heels |  |  |  |  |  |
| Heel to toe (tandem) walking |  |  |  |  |  |
| Heel to toe (tandem) walking backwards |  |  |  |  |  |
| Walking backwards |  |  |  |  |  |
| Walking sideways – cross over |  |  |  |  |  |
| Walking sideways – side steps |  |  |  |  |  |
| Stair walking |  |  |  |  |  |
| Walking and changing directions |  |  |  |  |  |
| Walking with frequent starts and stops |  |  |  |  |  |
| Walking with head turns |  |  |  |  |  |
| Walking in different directions |  |  |  |  |  |
| Walking and picking up objects |  |  |  |  |  |
| Walking while talking |  |  |  |  |  |
| Walking while balancing object |  |  |  |  |  |
| 1. **Yoga** |  |  |  |  |  |
| Mountain pose |  |  |  |  |  |
| Chair pose |  |  |  |  |  |
| Locust pose |  |  |  |  |  |
| Warrior 1 pose |  |  |  |  |  |
| Warrior 2 pose |  |  |  |  |  |
| Awkward pose |  |  |  |  |  |
| Tree pose |  |  |  |  |  |
| Eagle pose |  |  |  |  |  |
| Sun salutation |  |  |  |  |  |
| Cobra pose |  |  |  |  |  |
| Table top pose |  |  |  |  |  |
| Sphinx pose |  |  |  |  |  |
| Seal pose |  |  |  |  |  |
| Happy Baby pose |  |  |  |  |  |
| Refined Cobbler pose |  |  |  |  |  |
| Downward Dog pose |  |  |  |  |  |
| Hero pose |  |  |  |  |  |
| Fish pose |  |  |  |  |  |
| Moonflower |  |  |  |  |  |
| Sunflower |  |  |  |  |  |
| Prayer to heart center |  |  |  |  |  |
| Monkey pose |  |  |  |  |  |
| Triangle pose |  |  |  |  |  |
| Reverse triangle pose |  |  |  |  |  |
| Pigeon pose |  |  |  |  |  |
| Cat/Cow |  |  |  |  |  |
| Pyramid pose |  |  |  |  |  |
| Other poses (DESCRIBE IN NOTES) |  |  |  |  |  |
| 1. **Tai chi** |  |  |  |  |  |
| 24-form Yang style |  |  |  |  |  |
| 18-form style |  |  |  |  |  |
| 12-form Sun style |  |  |  |  |  |
| 10-form Sun style |  |  |  |  |  |
| 8-form style |  |  |  |  |  |
| Chen style Evidence Based Tai Chi |  |  |  |  |  |
| Other: ___________________ |  |  |  |  |  |

| **CATEGORY** | **EXERCISE** | **No – not prescribed** | **Yes- sometimes** | **Yes- most of the time** | **IF YES** | |
| --- | --- | --- | --- | --- | --- | --- |
|  |  |  |  |  | **Yes – some participants** | **Yes – all participants** |
| 1. **Strength training** |  |  |  |  |  |  |
| **Standing** |  |  |  |  |  |  |
| **Seated** |  |  |  |  |  |  |
| **Lying** |  |  |  |  |  |  |
| *Chest* | Push-ups |  |  |  |  |  |
|  | Chest fly |  |  |  |  |  |
|  | Chest press |  |  |  |  |  |
| *Shoulders* | Overhead press |  |  |  |  |  |
|  | Deltoid lateral raise |  |  |  |  |  |
|  | Deltoid front raise |  |  |  |  |  |
| *Arms* | Bicep curls |  |  |  |  |  |
|  | Tricep extension |  |  |  |  |  |
| *Back* | Back extension |  |  |  |  |  |
|  | Deadlift |  |  |  |  |  |
|  | Single-leg deadlift |  |  |  |  |  |
|  | Rows |  |  |  |  |  |
|  | Bent-over row |  |  |  |  |  |
|  | Upright row |  |  |  |  |  |
| *Legs* | Side lunge |  |  |  |  |  |
|  | Front lunge |  |  |  |  |  |
|  | Squats |  |  |  |  |  |
|  | Hip abduction |  |  |  |  |  |
|  | Knee extension |  |  |  |  |  |
|  | Hamstring curls |  |  |  |  |  |
|  | Step-up |  |  |  |  |  |
|  | Resisted dorsiflexion |  |  |  |  |  |
| *Core* | Bird-dog |  |  |  |  |  |
|  | Dead bug |  |  |  |  |  |
|  | Ab Crunch |  |  |  |  |  |
|  | Oblique side bends |  |  |  |  |  |
|  | Plank |  |  |  |  |  |
|  | Side plank |  |  |  |  |  |
|  | Hip bridge |  |  |  |  |  |
|  | Suitcase carry |  |  |  |  |  |
| *Other* | Other (DESCRIBE IN FIELD NOTES) |  |  |  |  |  |

**NOTES:**______________________________________________________________________________________________________________________________________________________

1. In your opinion, how effective is your program at improving confidence with moving in your participants?
   - 1. Not at all effective
     2. Somewhat ineffective
     3. Neutral
     4. Somewhat effective
     5. Very effective
     6. Don’t know
2. In your opinion, how effective is your program at decreasing fear of falling in your participants?
   - 1. Not at all effective
     2. Somewhat ineffective
     3. Neutral
     4. Somewhat effective
     5. Very effective
     6. Don’t know
3. In your opinion, how effective is your program at improving balance in your participants?
   - 1. Not at all effective
     2. Somewhat ineffective
     3. Neutral
     4. Somewhat effective
     5. Very effective
     6. Don’t know
4. In your opinion, how effective is your program at reducing falls in your participants?
   - 1. Not at all effective
     2. Somewhat ineffective
     3. Neutral
     4. Somewhat effective
     5. Very effective
     6. Don’t know

**Assessment tools/measures used**

1. Do you conduct any evaluations, assessments, or established measures in your program?
   - 1. Yes, at the beginning of the program only
     2. Yes, at the end of the program only
     3. Yes, at the beginning and end of program
     4. Yes, sometimes
        1. Why do you sometimes perform them? _______________________
     5. No

If no, why not? ______________________________________________

1. If you answered yes to the above question, which evaluations, assessments, and/or measures do you use? (circle)

| Berg Balance Scale (BBS) |
| --- |
| Balance Evaluation Systems Test (BESTest), or Mini-BESTest or Brief BESTest |
| Clinical Test of Sensory Integration in Balance (Foam & Dome) |
| Community Balance and Mobility Scale |
| Dynamic Gait Index (DGI) |
| Fall Risk for Older People in the Community (FROP-Com) Screen |
| Fullerton Advanced Balance (FAB) Scale |
| Functional Reach |
| Maximal Step Test |
| Movement Observation |
| Romberg’s Test |
| Scott Fall Risk Screen (SFRS) |
| Single Leg Stance |
| Tandem Standing/Walking (independent of another test) |
| Timed Up & Go (TUG) |
| Tinetti: Performance-Oriented Mobility Assessment (POMA) |
| 5 Step Test |
| 30 Second Sit-to-Stand |
| Other (specify): |
| Other (specify): |
| Other (specify): |

**Falls Prevention Resources**

1. Are you familiar with any local or national resources regarding fall prevention?
   - 1. No
     2. Yes, describe: _____________________________________
2. The Winnipeg Regional Health Authority has created a variety of fall prevention “Staying on Your Feet” resources. Are you familiar with this series of fall prevention resources?
   - 1. No
     2. Yes
     3. Select all that apply:

- Educational booklet (Take Action to Prevent Falls)
- Checklist (To Help You Prevent Falls)
- Fact Sheet
  - Improve Your Balance and Strength
  - Proper Footwear Can Help Prevent Falls
- Poster
  - Exercise for Strength and Balance
  - Vitamin D Prevents Falls and Injuries
  - Some Medication Can Put You At Risk of Falling
  - Proper Footwear Can Help You Prevent a Fall
- Preventfalls.ca website
  - Exercise component
  - Online home safety assessment
- Community Presentation (Staying on Your Feet)

**Key informant information**

1. What is your title or role in your organization?
   - 1. Director
     2. Manager
     3. Exercise leader
     4. Other, specify _____________________________________
2. How many years of experience have you had in this role?
   - 1. 0-1
     2. 2-3
     3. 4-5
     4. 5+
3. What is your educational background/training? (PROMPT)
   - 1. Exercise Physiology/Kinesiology
     2. Physiotherapy
     3. Nursing
     4. Other, specify ____________________________________
4. Have you received any specific training or education in falls prevention?
   - 1. No
     2. Yes, please describe: __________________________________________)
5. Have you had any experience in providing falls prevention training and education?
   - 1. No
     2. Yes
6. If yes, for how many years?
   - - - 1. 0-1
         2. 2-3
         3. 4-5
         4. 5+
7. How knowledgeable are you are with respect to understanding falls prevention exercises?
8. Not at all knowledgeable
9. A little knowledgeable
10. Somewhat knowledgeable
11. Very knowledgeable
