# Supplementary material for: To what extent do older adult community exercise programs in Winnipeg, Canada address balance and include effective fall prevention exercise? A descriptive self-report study
Source: BMC Geriatr. 2019 Jul 29;19:201. doi: 10.1186/s12877-019-1224-x (PMC6664743; doi:10.1186/s12877-019-1224-x)
Supplement: Supplementary file 3 — Components of balance operational definitions. Doc (DOCX 17 kb) [file 12877_2019_1224_MOESM3_ESM.docx]

**Additional file 3: Components of balance operational definitions**

| **Domains in Systems Framework for Postural Control** | **Scoping Review Adaptation of Component of Balance and Operational Definition** |
| --- | --- |
| 1. Biomechanical constraints: degrees of freedom, strength, limits of stability | 1. Functional stability limits: Ability to move the center of mass as far as possible in the anteroposterior or mediolateral directions within the base of support  2. Underlying motor systems: eg, strength and coordination  3. Static stability: Ability to maintain position of the center of mass in unsupported stance when the base of the support does not change (may include wide stance, narrow, 1-legged stance, tandem—any standing condition) |
| 2. Orientation in space: perception of gravity, verticality | 4. Verticality: Ability to orient appropriately with respect to gravity (eg, evaluation of lean) |
| 3. Movement strategies: reactive, anticipatory, voluntary | 5. Reactive postural control: Ability to recover stability after an external perturbation to bring the center of mass within the base of support through corrective movements (eg, ankle, hip, and stepping strategies)  6. Anticipatory postural control: Ability to shift the center of mass before a discrete voluntary movement (eg, stepping-lifting leg, arm raise, head turn) |
| 4. Control of dynamics: gait, proactive | 7. Dynamic stability: Ability to exert ongoing control of center of mass when the base of the support is changing (eg, during gait and postural transitions) |
| 5. Sensory strategies: integration, reweighting | 8. Sensory integration: Ability to reweight sensory information (vision, vestibular, somatosensory) when input altered |
| 6. Cognitive processing: attention, learning | 9. Cognitive influences: Ability to maintain stability while responding to commands during the task or attend to additional tasks (eg, dual-tasking) |

Retrieved with permission from Sibley KM, Beauchamp MK, Van Ooteghem K, Straus SE, Jaglal SB. Using the Systems Framework for Postural Control to Analyze the Components of Balance Evaluated in Standardized Balance Measures: A Scoping Review. Archives of Physical Medicine and Rehabilitation. 2015;96(1):122-32.e29.
